# Importance of Routine Laboratory Investigations Before Elective Surgery

**DOI:** 10.15190/d.2020.11

**Published:** 2020-09-30

**Authors:** Ashish K. Kannaujia, Amrita Gupta, Shiva Verma, Uma Srivastava, Rudrashish Haldar, Soni Jasuja

**Affiliations:** ^1^Department of Anesthesiology, Sanjay Gandhi Post Graduate Institute of Medical Sciences, Lucknow, India; ^2^Department of Anaesthesiology, Sarojani Naidu Medical College, Agra, India; ^3^Department of Microbiology, Sarojani Naidu Medical College, Agra, India

**Keywords:** Pre-operative investigation, investigation cost, post-operative outcome, post-operative complications.

## Abstract

BACKGROUND AND AIMS: Certain routine pre-operative laboratory investigations are performed in all patients before elective surgeries. We conducted this study to assess the value of routine pre-operative tests in the ASA (American Society of Anesthesiologists) Grade I and II adults undergoing elective surgery and their influence in the conduct of anaesthesia together with the costs incurred on unwarranted tests. METHODS: A total of 1271 patients posted for elective surgery under anaesthesia were recruited. Each patient attended the Pre-Anaesthetic Checkup Clinic and underwent clinical evaluation and investigations according to institutional policy. Demographic data and other characteristics were recorded, along with the results of laboratory test, any peri-operative intervention done as a result of abnormality and the cost incurred on tests. RESULTS: Majority of the patients belonged to ASA status I (74%) and underwent moderately invasive surgery (78%). The total number of routine investigations performed was 8015. Of these, 351 (4.37%) tests had abnormal results. Amongst these 333 (4.15%) abnormalities were suspected clinically and peri-operative intervention was only performed in 0.43% of patients. Anemia was the most common abnormal finding. Abnormal blood glucose was detected in 6 patients who were not clinically suspected. Abnormal electrocardiograph (ECG) was found in 54 patients. However, the intervention was required only in 13 patients. No intervention was required because of abnormal findings of the chest X-Ray. In total cost of investigations, only 6.9% was contributed by abnormal investigations and the rest was spent on the normal tests. CONCLUSION: The incidence of tests with abnormal results was very low in our study, and less than 1% of the patients with abnormal tests required changes in their peri-anaesthetic management. No major complications were seen in any patient with normal or abnormal test results. Most of the expenses (93%) were related to the normal test, which did not contribute to the perioperative management, safety and outcome of the patient. Thus, pre-operative investigations should be judiciously advised to avoid inconvenience, surgical delays and escalation of the costs of surgical care.

## INTRODUCTION

Pre-operative laboratory testing is considered an integral part of pre-anaesthetic evaluation. Pre-operative tests can be categorized as discretionary or indicated, and routine or screening tests^1^. Indicated tests are defined as tests performed for a specific indication or purpose, based on the history and clinical examination of the patient and these tests are generally well accepted (e.g. to confirm a clinical diagnosis). The routine tests are defined as tests used to reveal unsuspected disease (e.g. routine blood investigations, electrocardiography (ECG) or chest X-ray). The usefulness of routine tests is being questioned, because they rarely detect unsuspected disease, and abnormality in the tests does not influence peri-operative management or outcome. The unnecessary tests escalate the cost of surgical care without providing any safety for medico-legal liability^1-7^.

Even though routine pre-operative tests play no beneficial role in normal healthy surgical patients, ordering a battery of routine tests is widely practiced. In our institution, a number of pre-operative tests are performed without any consideration of patient’s age, medical history, severity of surgery, or considerations of cost.

This prospective cross-sectional study was aimed to determine the incidence of abnormal results and cost incurred on routine laboratory tests of otherwise healthy surgical patients. We also investigated if abnormal results influenced the decision regarding any change in peri-anaesthetic management and the outcome.

## MATERIALS AND METHODS

This observational study was conducted over a period of 18 months, after approval from Institutional Ethics Committee and obtaining written consent from the patients. Patients of either sex, aged 18 to 80 years, belonging to American Society of Anesthesiologists (ASA) status I and II and who attended Pre-Anaesthetic Checkup Clinic and were scheduled for elective orthopaedic, general surgical, gynaecologic, urologic, otorhinolaryngological or ophthalmic surgeries under general, regional or combined general-regional anaesthesia were recruited. Patients less than 18 years old or more than 80 years of age, being operated under local anaesthesia, belonging to ASA grade III or IV, or undergoing emergency surgery, were excluded from the study.

The recruitment of patients was based on a non-probability consecutive sampling technique. Each patient attended the Pre-Anesthetic Checkup clinic where thorough clinical evaluation (history taking and physical examination) was done by post-graduate residents (with more than one year of experience) under the supervision of a consultant anaesthesiologist. Routine blood investigations were sent according to institutional policy and included complete blood count (CBC), blood urea and serum creatinine in all patients, before sending the patient for Pre-Anesthetic Checkup. Fasting or random blood glucose was determined in patients above 40 years of age, ECG was performed in patients above 50 years of age and chest X-ray was used if required.

A specially designed proforma was filled for each patient. This included age, sex, ASA status, existing co-morbidity, type of surgery and anaesthesia, results of laboratory tests and any peri-operative intervention performed as a result of abnormality in the investigations. Peri-operative intervention was defined as referral to specialist, repeat or new tests ordered, postponement of surgery or change in anaesthetic plan. Any peri-operative complications related to abnormal test result were also noted.

The numerical value of each test was labelled as abnormal when its value fell outside the normal range as determined by the stated reference range printed on the form provided by the institutional laboratories. In addition, whether any test result revealed any disease which was not suspected clinically was also noted.

The results were assessed using descriptive statistics. Each test’s cost was calculated based on the price list provided by the institute’s laboratory.

## RESULTS

A total of 1271 patients were recruited in this study. As observed in Table 1, the age of the patients ranged between 19-78 years (47.28±18 years, mean ±SD) and there were 684 (54%) women as compared to men (587). The majority of the patients belonged to ASA status I (74%) and 78% underwent moderately invasive surgery. A total of 8015 investigations were performed in 1271 patients and 351 (4.37%) tests revealed abnormalities. These abnormalities were suspected in 333 (4.15%) patients during Pre-Anesthetic Checkup. Peri-operative interventions were performed in 35 (0.43%) patients due to abnormal test results. Most of the interventions were due to the low haemoglobin and abnormalities in ECG (Table 2 and Table 3).

**Table 1:** Patient demographics and other features SD - standard deviation.

Age in years	Range and mean±SD	19-45 years - 744 (58%); 45-60 years - 351 (28%); 60-78 years - 176 (14%); Mean±SD - 47.28±18
Gender	Female/Male	684/587
ASA Grade	Grade I	941 (74%)
	Grade 2	330 (26%)
Type of surgery	Orthopaedic	452
	General surgery	395
	Gynaecologic	317
	Urologic	87
	Others	20
Surgical Grade	Grade I	278 (22%)
	Grade 2	993 (78%)
Smoking	Smoker	165
Alcohol use	Alcohol use	192
Comorbidities	Hypertension	55 (4.3%)
	IHD	16 (1.3%)
	COPD	14 (1.1%)
	Diabetes	09 (0.7%)
	Thyroid Disorder	06 (0.47%)
	>1 comorbidity	12 (0.94%)

**Table 2:** Details of cost incurred by pre-operative investigations Interv – interventions; * - data expressed as number and percentage (%); # - cost is given in INR (Indian Rupees), as per institutional laboratory rates; TLC - total leucocyte count; DLC - differential leucocyte; ECG - Electrocardiography, USD - US Dollars.

Investigation	Total number of tests done	Number of abnormal tests* (%)	Number of patients with suspected abnormality* (%)	Number of interv. done* (%)	Cost per test (INR)	Cost of tests with normal results (INR)	Cost of tests with abnormal results (INR)
Haemoglobin	1271	197(15.49%)	222 (17.46%)	16(1.25%)	10	10740	1970
TLC/DLC	1271	32 (2.51%) /11 (0.86%)	0	0	10	12390	320
Platelet count	1271	06 (0.47%)	0	0	10	12650	60
Blood glucose	995 (78.28%)	15 (1.50%)	9 (0.90%)	5(0.50%)	10	9800	150
Blood urea	1271	06 (0.47%)	0	0	15	1897	90
Creatinine	1008 (79.30%)	03 (0.29%)	5	1(0.09%)	15	15045	45
ECG	583 (45.86%)	54 (9.26%)	102 (17.49%)	13(2.29%)	100	52900	5400
X-Ray Chest	345 (27.14%)	27 (7.82%)	12 (3.47%)	0	150	47700	4050
Total	8015	351 (4.37%)	333 (4.15%)	35(0.43%)	-	163122 (93.10%) USD 2240	12085 (6.90%) USD 165

**Table 3:** Details of Perioperative Interventions in Patients with Abnormal Investigation Results Table caption

Investigations	Abnormality	Interventions performed	Number of patients
Haemoglobin concentration	Low	Blood transfusion before surgery	3
		Arrangement of blood products before surgery	9
		Iron therapy and postponement of surgery	4
Creatinine and Urea	Raised	Advised to follow up with a nephrologist after surgery and discharge	1
Blood sugar	High	Referral to a specialist	5
X-Ray Chest	Prominent broncho-vascular markings, healed tuberculosis, early bronchiectatic changes, mild cardiomegaly	None	-
ECG	Sinus tachycardia (14 patients) Poor progression of R wave (13 patients) Low voltage complex (10 patients) Old myocardial infarction (7 patients) ST changes in one lead (4 patients) Right or left axis deviation (6 patients)	Referral to cardiologist	13

The most common abnormality observed in laboratory tests was low haemoglobin. Overall, 197 (15.5%) patients had low haemoglobin level (<10gm/dl) and it was more common in females (23% versus 10%, in females versus males) (Table 2). Peri-operative interventions (Table 3) were required in 16 patients: in the form of perioperative blood transfusion in 3 patients with pelvis fracture, with arrangement of blood products before surgery in 9 patients and with iron therapy and postponement of surgery in 4 patients (Table 3).

White blood count (WBC) was marginally beyond the reference range in 32 (2.5%) patients. No intervention was performed in the form of referral to specialist, further investigation, optimisation, change in plan of anesthesia or postponement of surgery in any patient. All the patients underwent surgeries without any subsequent complications. Differential white cell count was abnormal in 0.9% (11) patients but no change in peri-operative anaesthetic management was done. Platelet count was slightly below the reference range in 6 asymptomatic patients and they were operated without any complications.

Abnormal fasting/random blood glucose was detected in 15 (1.18%) patients. Out of these, 9 were known diabetics who were controlled on oral hypoglycaemic drugs or insulin. For other patients with high blood sugar action, 5 patients were referred to specialists and one patient showed marginally raised blood sugar and so no further action was needed. Clinically significant elevation of creatinine (1.9 mg/dl) was present in 1 patient and border line elevation was observed (1.3 mg/dL) in 2 patients. The patient with raised creatinine was hypertensive and diabetic posted for tibial interlocking. This patient was operated under spinal anaesthesia with no peri-anaesthetic complication with the advice to consult her physician after discharge. Blood urea was marginally raised in 6 patients, but no further investigation or specialist consultation was required.

ECG was performed in 583 (45.86%) patients with abnormal findings in 54 (9.26%) patients. Interventions in the form of referral to cardiologist were recommended for 13 (2.29%) patients. Chest X-ray was performed in 345 (27.14%) patients, with abnormal findings seen in 27 (7.82%) patients. Intervention in the form of further investigation, consultation to specialist or postponement of surgery was not required in any patient. Details of abnormalities detected in chest X-ray and ECG and peri-operative interventions done are given in Table 3. No major complication was seen in any patient with normal or abnormal test results.

The total cost of the performed tests was Indian Rupee (INR) 175207 (USD 2400) out which abnormal test contributed only 6.90% (INR 12085 = USD 165) and normal test contributed 93.10% (INR 163122 = USD 2240).

## DISCUSSION

The results of this study demonstrated that performing large number of pre-operative routine tests is inappropriate and unnecessarily increases the costs. Out of 8015 routine laboratory tests, only 351 (4.37%) tests revealed abnormal results, of which abnormality was suspected clinically, based on history and examination in 333 cases (4.15%). In patients with abnormal test results peri-operative intervention was required in only 35 (0.43%) cases. Amongst abnormal test results low haemoglobin level was the most common abnormality (15.49%), as also reported from other developing countries^8^, while most studies from Western world found anaemia in less than 3% of patients^9^. Generally, low haemoglobin contributes little to patient’s surgical management or morbidity^10^and most cases of anaemia which are significant enough for patient’s management can be detected clinically^11^. In this study anaemia was also clinically diagnosed in 222 patients and confirmed by laboratory investigation in 197 cases, thus showing a good clinical correlation.

Total and differential leukocyte count is rarely deranged in normal elective surgical patients and it alone does not affect patient management as evidenced in this study. Likewise, renal functions quantified by urea and creatinine, during pre-operative period, showed marginally high values in few patients, motivating no alteration in anaesthetic plan, in peri-operative period. The abnormal blood glucose was found in 15 patients, of which 9 were known diabetics and in six patients (0.5%) it was not clinically suspected.

ECG and chest X-ray are the two frequently performed screening tests^9^ that unnecessarily increase the cost of surgical care without much benefit. ECG abnormalities were detected in 9.26 % of the patients and intervention was needed only in 13 patients. Similar to our study, Turnbull and Buck^5^found abnormality in 16% of ECGs of healthy patients without any peri-operative consequences. Parez et al.^9^ reported 10.4% abnormal ECG, from which 5.6% were unexpected and only 0.46% required change in perioperative management. Despite some abnormality in chest X-ray of 27 patients, no intervention was required for abnormal findings of chest X-ray.

Pre-operative tests should be ordered with an intent to confirm a suspicion of an ailment backed by reasonable clinical basis and to determine judicious anaesthetic management and timing and to gauge the risk of peri-operative complications. If a test fails to meet above criteria, it will lead to an increase in unnecessary costs, inconvenience and surgical delays^12^.

Many retrospective and prospective studies have been done to investigate the usefulness of routine pre-operative laboratory tests^1,5,6,8,9,13-15^. These studies revealed that 70-90% of the routine blood tests were unnecessary^16^and less than 5% of tests showed abnormality^1,13^. The findings of the present study are in accordance with the above studies. A study by Guttikinda et al.^17^ compared the cost of pre-operative investigations ordered in their institute with NICE guidelines 2016, they found that indicated tests as per the guidelines constituted only 7% of cost of all performed tests, with the remaining cost being due to unindicated tests^17,18^. In this study the abnormal tests also contributed only a fraction (6.9%) of the total cost of the investigations. Most of the expenses (93.1%) were on the tests which were neither required on the basis of clinical evaluation nor altered the patient’s peri-anaesthetic management.

The test abnormalities rarely influenced patient’s anaesthetic management^1,5,7,9,13^ or post-operative outcome^1,5,11^, a finding in agreement with this study.

It has been shown that unnecessary tests may cause harm to patients^1,19^and the chances of getting false positive results^13,20-22^ increases with the increasing in the number of performed tests. Elimination of unnecessary tests could eliminate the surgery delay^22^and lower the risk of unnecessary follow up of false positive or false negative tests^3,16^. Cost containment of health care is an additional benefit^1,4,7,23^.

The results of the present study are in general agreement with the above observations. When a laboratory test shows an abnormal result with no significant clinical implication, the clinician omits the result, and goes ahead with anaesthesia and surgery without any intervention (Table 3). Thus, the indiscriminate routine tests serve no benefit or utility^13^. Although the value of pre-operative screening tests has been questioned repeatedly over the last three decades, physicians continue to perform it. The reason for this is not clear^24,25^. Fear for litigation^19,20,26^, concerns about surgical delays^22,25-27^, institutional policies^20,26^, complex health care environment^3^ and difficulty in changing ingrained habits^16,20^are some of the proposed causes. The litigation potential is considered an impediment in publication of pre-operative test guidelines itself^28^. These guidelines also leave the final judgement on requirement of certain tests on the treating clinician.

Fear of litigation is a real concern among clinicians. They are worried that, if they are not doing routine tests, they will be held responsible in case of an adverse event during anaesthesia^20,26,29^. Although the practice of failing to pursue an abnormal test result, probably leaves the clinician open to more medico-legal risk than if the test wasnot ordered in the first place^20^.

To conclude, indiscriminate use of screening laboratory tests for every patient without consideration of health status of patients and type of surgery, serves no benefit in peri-operative management of otherwise healthy surgical patients.

Practice advisory concluded that routine testing does not make a valuable contribution to pre-operative evaluation, while indicated tests may help in peri-operative decision making.

The decision regarding patient’s fitness for anaesthesia and surgery can be accurately based on patient’s clinical history and examination. These findings should guide the selection of tests. Significant financial benefit can be realized by ordering selected tests based on the history and clinical examination of the individual patient.

## CONCLUSION

Pre-operative tests should be ordered based on the history and physical examination of the individual patients to confirm a suspected disease, to optimize the patient management, to decide anaesthetic management and to predict peri-operative complications. Incidence of abnormal tests is very low in ASA-I and II patients requiring elective surgery and the patients rarely require change in peri-anesthetic management due to abnormal test results. The routine tests besides escalating cost of surgical care, serve no benefit to these patients. Pre-operative testing-based on the clinical condition of the individual patient will give significant financial benefits without compromising patient safety and quality of healthcare.
